# The impact of peer pressure on cigarette smoking among high school and university students in Ethiopia: A systemic review and meta-analysis

**DOI:** 10.1371/journal.pone.0222572

**Published:** 2019-10-11

**Authors:** Cheru Tesema Leshargie, Animut Alebel, Getiye Dejenu Kibret, Molla Yigzaw Birhanu, Henok Mulugeta, Patricia Malloy, Fasil Wagnew, Atsede Alle Ewunetie, Daniel Bekele Ketema, Alehegn Aderaw, Moges Agazhe Assemie, Getachew Mullu Kassa, Pammla Petrucka, Amit Arora

**Affiliations:** 1 College of Health Sciences, Debre Markos University, Debre Markos, Ethiopia; 2 Department of Nursing, College of Nursing, University of Saskatchewan, Regina, Canada; 3 Colleges of Nursing, University of Saskatchewan, Saskatoon, Canada; 4 School of Life Sciences and Bioengineering, Nelson Mandela African Institute of Science and Technology, Arusha City, Tanzania; 5 School of Science and Health, Western Sydney University, Penrith, NSW, Australia; 6 Translational Health Research Institute, Western Sydney University, Penrith, NSW, Australia; 7 Discipline of Child and Adolescent Health, Sydney Medical School, Faculty of Medicine and Health, The University of Sydney, Westmead, NSW, Australia; 8 Oral Health Services, Sydney Local Health District and Sydney Dental Hospital, NSW Health, Surry Hills, NSW, Australia; University of Mississippi Medical Center, UNITED STATES

## Abstract

**Background:**

Cigarettes and their by-products (i.e., smoke; ash) are a complex, dynamic, and reactive mixture of around 5,000 chemicals. Cigarette smoking potentially harms nearly every organ of the human body, causes innumerable diseases, and impacts the health of smokers and those interacting with the smokers. Smoking brings greater health problems in the long-term like increased risk of stroke and brain damage. For students, peer pressure is one of the key factors contributing to cigarette smoking. Therefore, this systematic review and meta-analysis assessed the impact of peer pressure on cigarette smoking among high school and university students in Ethiopia.

**Methods:**

An extensive search of key databases including Cochrane Library, PubMed, Google Scholar, Hinari, Embase and Science Direct was conducted to identify and access articles published on the prevalence of cigarette smoking by high school and university students in Ethiopia. The search period for articles was conducted from 21^st^ September, 2018 to 25^th^ December 25, 2018. All necessary data were extracted using a standardized data extraction checklist. Quality and risk of bias of studies were assessed using standardized tools. Heterogeneity between the included studies was assessed using Cochrane Q-test statistic and *I*^*2*^ test. To estimate the pooled prevalence of cigarette smoking, a random effects model was fitted. The impact of peer pressure on cigarette smoking was determined and was reported in Odds Ratio (OR) with 95% Confidence Interval (CI). Meta-analysis was conducted using Stata software.

**Results:**

From 175 searched articles, 19 studies fulfilled the eligibility criteria and were included in this study. The pooled prevalence of cigarette smoking among Ethiopian high school and university students was 15.9% (95% CI: 12.21, 19.63). Slightly higher prevalence of cigarette smoking was noted among university students [17.35% (95% CI: 13.21, 21.49)] as compared to high school students [12.77% (95% CI: 6.72%, 18.82%)]. The current aggregated meta-analysis revealed that peer pressure had a significant influence on cigarette smoking (OR: 2.68 (95% CI: 2.37, 3.03).

**Conclusion:**

More than one sixth of the high school and university students in Ethiopia smoke cigarette. Students who had peer pressure from their friends were more likely to smoke cigarette. Therefore, school-based intervention programs are needed to reduce the high prevalence of cigarette smoking among students in Ethiopia.

## Introduction

Smoking cigarettes yields a complex, dynamic and reactive mixture of around 5,000 chemicals [1–3]. Globally, it is one of the leading preventable causes of respiratory tract complications, disability, and early deaths related to complications [4–7]. It accounts for six of the eight leading causes of morbidity and mortality [5]. Essentially, it is a legal drug that kills many of its users when used exactly as intended by manufacturers. Currently, the World Health Organization (WHO) estimates that the use of both smoking and smokeless tobacco account for around 6 million deaths worldwide annually, of which 600,000 deaths were among non-smokers due to exposure to the smoke [8]. More than 30% of world’s adult population are consumers of tobacco, which leads to a warning that a billion people will die of adverse health effects related to the tobacco epidemic within the 21st century unless effective preventative measures are undertaken [3].

Smoking affects almost every organ in the human body (such as circulatory, respiratory, gastrointestinal and musculoskeletal systems), increases the risk for several diseases, and reduces the health of smokers in general [9, 10]. The key effect of smoking cigarettes is primarily on the lungs with approximately 85% of chronic obstructive pulmonary disease (COPD) and lung cancer and about 33% of other cancers (i.e., esophagus, oral cavity, uterus, stomach, and pancreas) related to smoking [9–11].

Normal adolescent developmental stage is affected by high level of peer pressure that can influence risk-taking behaviors including substance use [12]. Globally, especially in low- and middle-income countries, an estimated 80% of the one billion adolescent smokers are suffering from tobacco-related morbidity and mortality [7]. Cigarette smoking negatively influences the physical and mental health of an individual [13]. This is particularly true for high school and university students who already face major health challenges such as stress [14]. Smoking is also associated with poor educational performance, high-risk drinking behavior, illegal drug use, and high-risk sexual behaviors [14, 15]. Peer pressure is widely recognized as a crucial factor affecting young people's early experimentation with tobacco and their willingness to continue smoking [16]. Several students attending higher education institutions practice cigarette smoking for several reasons, such as a way to cope with stress [17]. Factors that contribute to the continued use of tobacco include being male, drinking alcohol, having a friend who drinks alcohol, having a friend who smokes, having family members who smoke and being older in age, to mention some [18].

In sub-Saharan Africa, the prevalence of smoking is increasing and is projected to continue to increase [19, 20]. The current data in the region reveals substantial variation in smoking rates among countries ranging from 1.8% in Zambia to 25.8% in Sierra Leone [21]. In Ethiopia, cigarette smoking is among one of the most commonly used substances, which leads to addiction [22]. It has deleterious effects on the health of the young users, significantly reduces academic performance in students and increases risk of contracting HIV and other sexually transmitted diseases. Several primary studies on the prevalence and associated factors of cigarette smoking among high school and university students have been conducted in Ethiopia [23–37]. According to earlier reviews of the literature, prevalence of smoking in Ethiopia ranges from 2.99% in Addis Ababa [38] to 28.6% in Hawassa and Jima University [30]. Therefore, this systematic review and meta-analysis aimed to review the pooled prevalence of cigarette smoking among high school and university students in Ethiopia and the impact of peer pressure on cigarette smoking among high school and university students in Ethiopia.

## Method and materials

This systematic review is based on the Preferred Reporting Items of Systematic Reviews and Meta-Analysis (PRISMA) checklist guidelines to ensure scientific rigor [39] (S1 Table). Prospective registration of systematic review and meta-analysis promotes transparency, helps reduce potential for bias, and improves review’s credibility. However, this meta-analysis and systematic review was not registered on the prosperous, and we have acknowledged this gap in the limitation section.

### Setting

This systematic review and meta-analysis reports data from Ethiopia. Ethiopia is located in the north-eastern part of the African continent or what is known as the “Horn of Africa”. The country is divided into nine regional states and two administrative cities [40] containing a total of 108,386,391 million population with a national density of 94 people per square kilometer, 2019 [41]. Ethiopia shares land borders with five countries: Sudan, Somalia, Djibouti, Eritrea, and Kenya [42].

### Inclusion and exclusion criteria

#### Eligibility criteria

This systematic review and meta-analysis included studies only conducted in Ethiopia that assessed the prevalence of cigarette smoking. Published articles were reviewed and rated for inclusion. Full articles were retrieved if a specific outcome of interest (smoking status) was defined. This review included all observational study designs (cross-sectional studies, case-control studies, and cohort studies). However, case reports or case series, duplicate reports, and inconsistent outcome measures were excluded. Moreover, we excluded articles that were published in a language other than English. Documents that were not accessible after contacting the principal investigator three times by email were also excluded. Articles that reported measures other than Relative Risk (RR) or equivalent values, or from which an Odds Ratio (OR) could not be calculated were also excluded from consideration, The eligibility criteria for each individual article were checked by three authors independently (CT, AA1, and AA2). If there was a disagreement between the two authors, a third person (UGM) resolved the disagreement. All reviewers came together in person and discussed the assessment results.

### Information sources

This systematic review and meta-analysis were conducted by considering all the available studies (both published and open grey reports), governmental and other stakeholder annual reports, and national surveys on children and adolescents which have data on cigarette smoking among high school and university students in Ethiopia. An extensive search was done from the following international databases, including Cochrane Library, PubMed, Google Scholar, Hinari, Embase, CINAHL, Web of Science, and Science Direct to access articles conducted on the prevalence of smoking cigarette. The following keywords “prevalence”, ("cigarette smoking" OR ("cigarette"[All Fields] AND "smoking"[All Fields]) OR "cigarette smoking"[All Fields]) AND substance[All Fields]) AND (high[All Fields] AND ("schools"[MeSH Terms] OR "schools"[All Fields] OR "school"[All Fields]) AND ("universities"[MeSH Terms] OR "universities"[All Fields] OR "university"[All Fields])) AND ("students"[MeSH Terms] OR "students"[All Fields]) AND ("Ethiopia"[MeSH Terms] OR "Ethiopia"[All Fields]) were used to obtain published articles. Boolean operators particularly pairing aspects of “OR” or “AND” were used as search terms to separate articles. The search for all articles was conducted from 21^st^ September, 2018 to 25^th^ December, 2018 (S2 Table).

### Data items

This systematic review and meta-analysis had two outcomes. The first outcome was the pooled prevalence of cigarette smoking among high school and university students in Ethiopia, which was calculated by dividing the number of smokers to the total students (sample size) multiplied by 100. The second outcome was the impact of peer pressure on cigarette smoking practice. We adjusted the effect size into Odd Ratio (OR) since all the studies were cross sectional and the appropriate effect size estimate for cross sectional design is OR to estimate the impact of peer pressure on cigarette smoking.

### Data extraction

The necessary data (primary author, publication year, region, study design, sample size, prevalence of cigarette smoking) were extracted from the eligible articles by two authors (CT, AA and AA1) independently using prepiloted data extraction format prepared in Microsoft^™^ Excel spreadsheet (S3 Table). Any disagreements between the three reviewers in the review process were discussed with the three reviewer team members (GD, DB and PM) until consensus was reached. Moreover, the data of kappa of agreement during the systematic searches was also used to solve the disagreements among two independent reviewers (CT and AA4). The kappa agreement was interpreted as less than chance agreement if less than 0, slight agreement if 0.01–0.20, fair agreement if 0.21–0.40, moderate agreement if 0.41–0.60, substantial agreement if 0.61–0.80 and moderate agreement if the kappa was 0.81–0.99 [43].

The four authors (CT, FW, MA and AA1) also independently extracted data on the association of cigarette smoking and peer pressure. If studies did not report OR, RR, or equivalent measures, raw data were screened to determine whether OR could be calculated. When the studies reported both the crude OR/RRs and the adjusted OR/RRs, the adjusted figures were extracted.

### Quality assessment of the included studies

We assessed the quality of the included studies according to the Newcastle-Ottawa Scale (NOS) [44] **(**S4 Table). The NOS has three main domains and uses a star-based grading system with each study scoring a maximum of 10 stars. The first domain focuses on the methodological quality of the study (sample size, response rate, and sampling technique) with the possibility of a five-star grading (1 = poor to 5 = excellent). The second domain of the tool deals with the comparability of the study cases or cohorts, with the possibility of two stars. The last domain deals with the outcomes and statistical analysis of the study with a possibility of three stars. Three authors (MA, UGM, and DB) independently assessed the quality of each included study using the NOS. Any disagreement between the three authors was resolved by requesting other two authors (MY and PP) to independently assess the methodological quality to reach a consensus. Finally, studies with stars of ≥ 7 out of 10 were considered to be of a high quality [45]. Moreover, we assessed the quality of each included articles using National Institutes of Health (NIH) (S5 Table) which is a more detail tool on quality assessment than NOS. The tool has 14 criteria to assess the article independently with a response of “Yes, No and Not Applicable”. Articles with NIH assessment result of 85% and more (that means number of articles with yes divided by total criteria minus not applicable) were considered as good quality.

### Risk of bias

For each included study, the risk of bias was assessed independently by two authors (UGM and CT). Risk of bias assessment was carried out using Holly 2012 tool which contain 10 recommended criteria for the internal and external validity tool [46]. This tool includes: representation of the population, sampling frame, methods of participants’ selection, non-response bias, data collection directly from subjects, acceptability of case definition, reliability and validity of study tools, mode of data collection, length of prevalence period; and appropriateness of numerator and denominator. Each item was classified as low and high risk of bias. Unclear assessment was classified as high risk of bias. The overall score of the risk of bias was then categorized according to the number of high risk item scores for bias per study: low (≤ 2), moderate (3–4), and high (≥ 5) (S6 Table).

### Statistical data analysis

Standard error for all included studies was computed using the binomial distribution formula. Heterogeneity across studies were assessed by determining the p-values of Cochrane Q-test and I^2^-test statistics [47]. For meta-analysis result with significant heterogeneity, univariate meta-regression was used to assess the source of heterogeneity across each study. A funnel plot was also used for visual assessment of the publication bias. Asymmetry of the funnel plot is an indicator of potential publication bias. Furthermore, Egger’s test was used to determine if there was significant publication bias, and a *p*-value less than 0.10 was considered to indicate the presence of significant publication bias [48]. We selected Egger`s test to assess the publication bias because, the value of Egger`s test is more specific than Begg`s test [49, 50]. We conducted the log relative risk to assess the effect of peer pressure on students’ cigarette smoking status. Furthermore, sensitivity analysis using a random effects model was performed to assess the influence of a single study on the pooled prevalence estimates. Subgroup analysis was used to minimize the random variations between the point estimates of the primary study subgroup, and analysis was done based on study settings (i.e., institution). Univariable meta-regression analysis was also conducted with year of publication and the outcome variable. All data manipulation and statistical analysis were performed using Stata^™^ software (Version 14; Stata Corp, College Station, TX).

## Results

**Fig 1:**
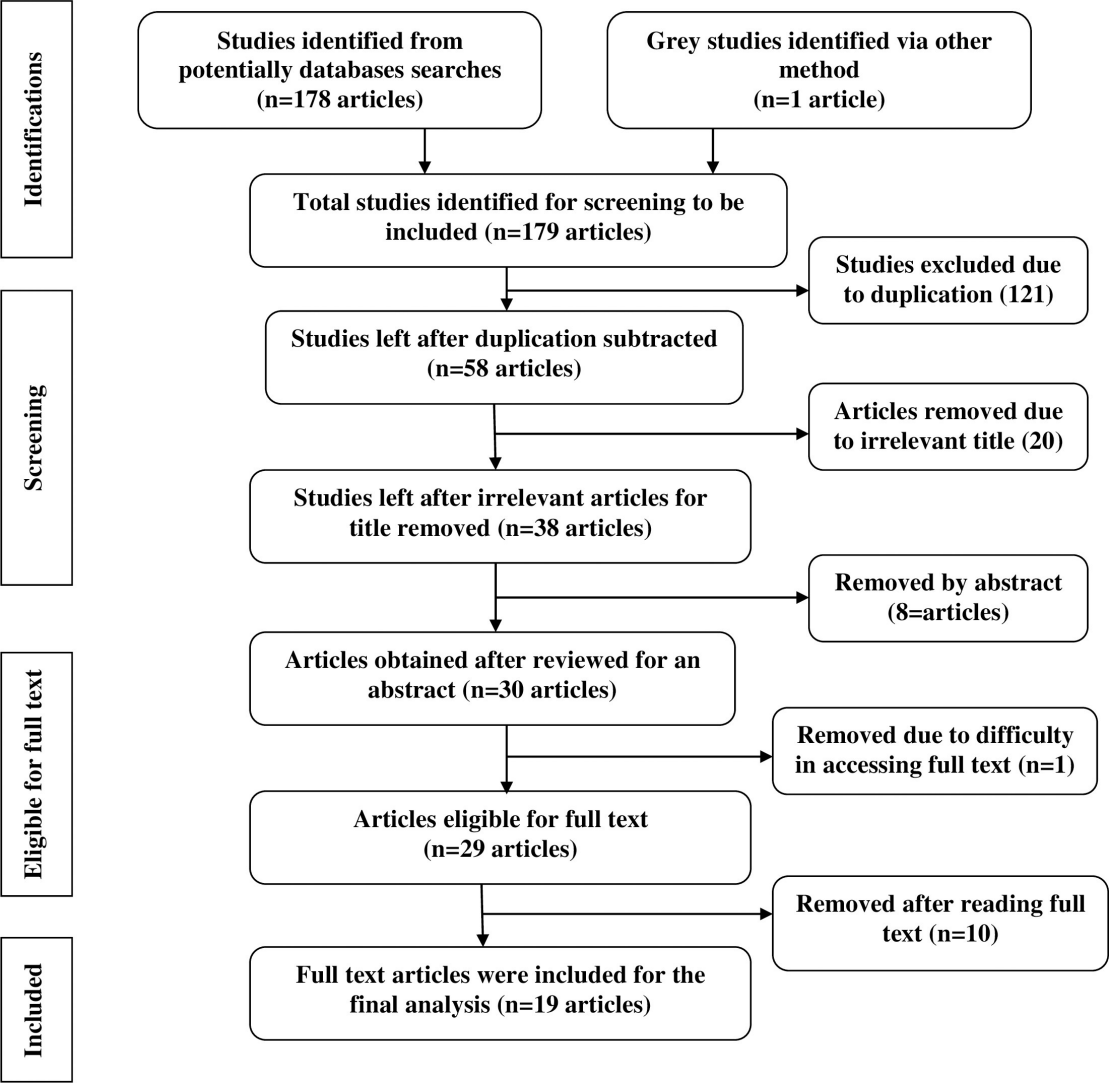
Describe the flow chart of selecting articles eligible for determining the prevalence of cigarette smoking: A systematic review and meta-analysis among high school and university students at Ethiopia.

The electronic database search identified a total of 179 published articles. Of these, 121 duplicate articles were removed. Furthermore, 28 articles were removed after reviewing the titles and the abstract as they were not relevant to the focus of the review. Finally, one article was excluded due to inaccessibility of the full text despite three requests to the primary author on data, and 10 articles were excluded after reviewing their full text. Finally, 19 articles met all the prior criteria and were included in this analysis (Fig 1).

### Overview of the original included articles

All of the 19 articles included in this study were published between 1999 to 2017 in peer-reviewed journals. A total of 16,486 study participants were included in this systematic review and meta-analysis. The smallest sample size was 155 from a study conducted at Bahir Dar University [36], and the largest sample size was 1,984 in a study conducted in Gondar Medical College, Amhara Region [34]. All included studies were cross-sectional in design. The characteristics of the studies included in this review are described in **(Table 1)**.

**Table 1:** Descriptive summary of 19 studies included in the meta-analysis of cigarette smoking and associated among high school and university students in Ethiopia.

Author	Publication year	Region	Study area	Study period	Mean age	Frequency of Male	Sample size	Prevalence (95% CI)
Ahmed [29]	2014	Oromia	Balie Preparatory	March 1–15, 2014	NA	125	220	11.36 (7.17, 15.56)
Andargachew [51]	2014	SNNPE	Hawassa University	July 2014	20.7(±1.49) years, (15–30 years)	419	586	14.85 (11.97, 17.73)
Andualem [35]	2014	Oromia	Haramaya University	Dec 2010 to Jan 2011	21 (SD±1.2) years	438	725	11.31 (9.00, 13.62)
Anteneh [37]	2014	Amhara	WHS	April 2015.	17.25 (SD±1.24) (14–19) years	358	651	22.89 (19.66, 26.12)
Ashete [23]	2017	Amhara	Woldia University	April 2015.	20.7 (SD ± 1.36) (18–25)years	454	655	7.94 (5.87, 10.01)
Ayalu [24]	2012	Harari	High school	April 12–26, 2010	16.4 (sd±1.60) (15–25)years	856	1721	12.43 (10.88, 13.99)
Emmanuel[38]	2007	Addis Ababa	High school	Not Reported	15 years.	787	1868	3.00 (2.22, 3.77)
Gezahegn[25]	2014	Oromia	Haramaya University	April 15–30, 2013	20.9 (SD = ±2.17 years)	777	1,022	22.02 (19.48, 24.56)
Girmay [26]	2014	Amhara	DMU	May 1–10, 2013	21.6 (SD ± 3.4) years	468	800	10.00 (7.92, 12.08)
Measho [28]	2013	Tigray	Axum university	April 2012	20.5 (SD ± 2.2) years	444	756	9.52 (7.43, 11.62)
Nebiyu [30]	2014	SNNPRE	HaU and JU	April 10–15,2014	NA	**793**	1673	28.63 (26.47, 30.80)
Tadele K [32]	2017	Somalia	Jigjiga University	Not Reported	NA	396	600	14.50 (11.68, 17.32)
Tadele E [31]	2014	Tigray	Mekelle University	March 2013	21.2 (±1.7 SD) years	96	197	28.93 (22.60, 35.27)
Tesfa [66]	2017	SNNPE	Wolaita Sodo University	Feb 10–20, 2015	21.18 (SD ± 1.79) years	482	725	19.86 (16.96, 22.77)
Tesfahun [11]	2013	Amhara	DMPTC	March 27, 2013	19.8 ± 2.1 years.	225	423	14.18 (10.86, 17.51)
Tiruwork[36]	2016	Amhara	Bahir Dar University	Not Reported	24.26 (SD±2.15) years	104	155	27.74 (20.69, 34.79)
Wakgari [33]	2011	Addis Ababa	Addis Ababa	June 2009	NA	426	622	9.00 (6.75, 11.25)
Yigzaw[27]	2002	Amhara	college students	January 2001	20.9 (SD±3.08) years	932	1103	13.15 (11.15, 15.14)
Zein [34]	1984	Amhara	Gondar Medical College	April 1983	NA	391	479	25.05 (21.17, 28.93)

### Quality assessment result of the included articles

The qualities of individual articles were assessed using different tools; namely NOS and NIH quality assessment tools. Accordingly, NOS assessment result all articles had good quality using the NOS criteria. However, when assessed using NIH quality assessment tool, 1 (5.3%) study [36] was categorized as poor and the rest [11, 15, 23–35, 37, 38, 51] were categorized as good quality (S5 Table).

### Kappa agreement

Disagreements between the two reviewers during data extraction process were assessed using the Kappa agreement. Therefore, a = 9 and b = 2 represent the number of times the two reviewers agreed while c = 1 and d = 7 represent the number of times the two reviewers disagree. If there are no disagreements, b and c would be zero, and the reviewers agreement (po) is 1, or 100%. If there are no agreements, a and d would be zero, and the reviewers agreement (po) is 0. Interobserver agreement was 68% that indicate a substantial agreement between the two main reviewers who extracted data.

### Risk of bias

Risk of bias was performed for each included study using the risk of bias assessment tool that includes ten different items [46]. From the 19 included studies, the risk of bias summary assessment revealed that 94.7% of the included studies had a low risk of bias [15, 23–35, 37, 38, 51] while only one (5.3%) of the included studies had a moderate risk of bias [36].

### Prevalence of cigarette smoking

**Fig 2:**
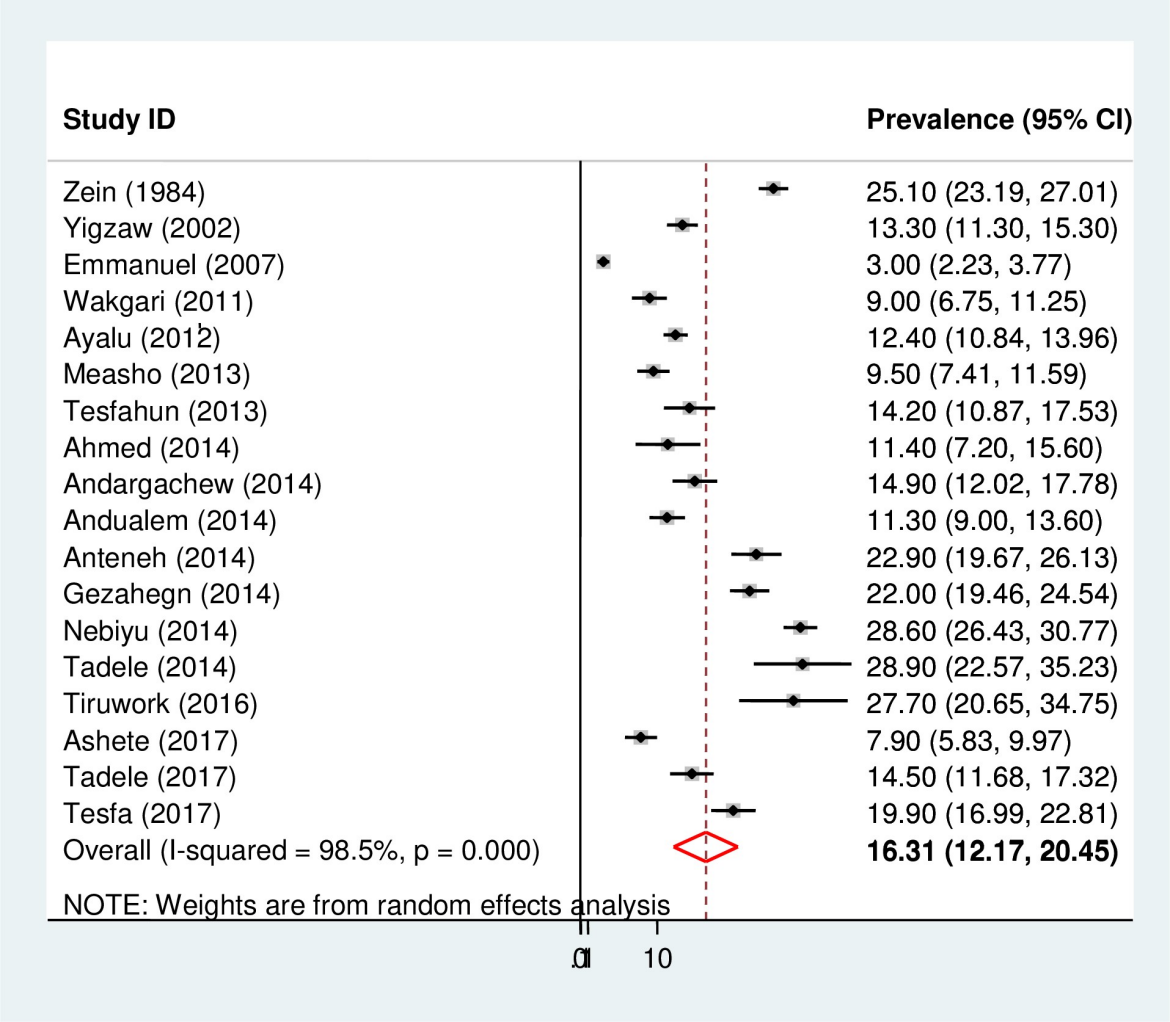
Forest plot of the pooled prevalence of cigarette smoking among high school and university students in Ethiopia.

The overall pooled prevalence of cigarette smoking in Ethiopia using the 19 studies was 16.31% (95% CI: 12.17, 20.45). A random-effects model was used because of the significant heterogeneity (*I*^*2*^ = 98.1%, p-value <0.001) across the studies (Fig 2). Additionally, univariate meta-regression analysis was conducted to identify possible sources of heterogeneity. The different covariates included in the analysis were publication year and sample size. However, none of these variables were found to be statistically significant.

**Fig 3:**
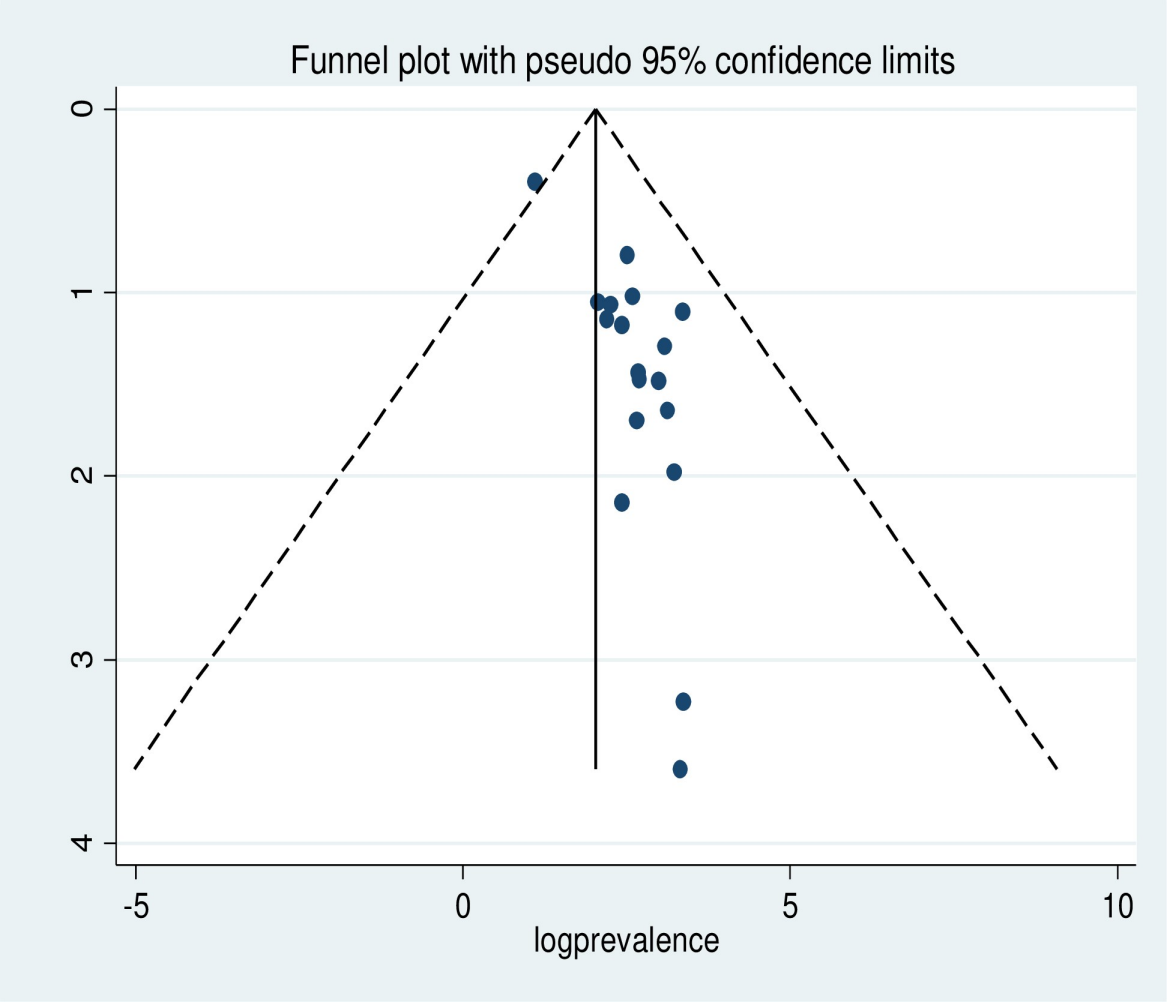
Funnel plot with 95% confidence limits of the pooled prevalence of smoking cigarette among high school and university students in Ethiopia.

The existence of publication bias was assured by funnel plot asymmetry. The funnel plot graph indicates that there is a significant variability within the findings of the 19 individual primary articles included in this meta-analysis (Fig 3). The publication bias checked by objective measurement namely Egger’s tests also showed a statistically significant publication bias (**Egger's test *p-value*** = ***0*.*001***). To handle the observed publication bias, we performed the trim and fill analysis, which is a nonparametric methods for estimating the number of missing studies that might exist and helps in reducing and adjusting publication bias in meta-analysis.

### Assessment of heterogeneity

We used *I*^*2*^ statistics to investigate the presence of variation across the included studies. Accordingly, the result of *I*^*2*^ statistics using a random effects model revealed a significant heterogeneity across the included studies ((I^2^ = 98.1%, *p-value <0*.*001*).

### Subgroup analysis

**Fig 4:**
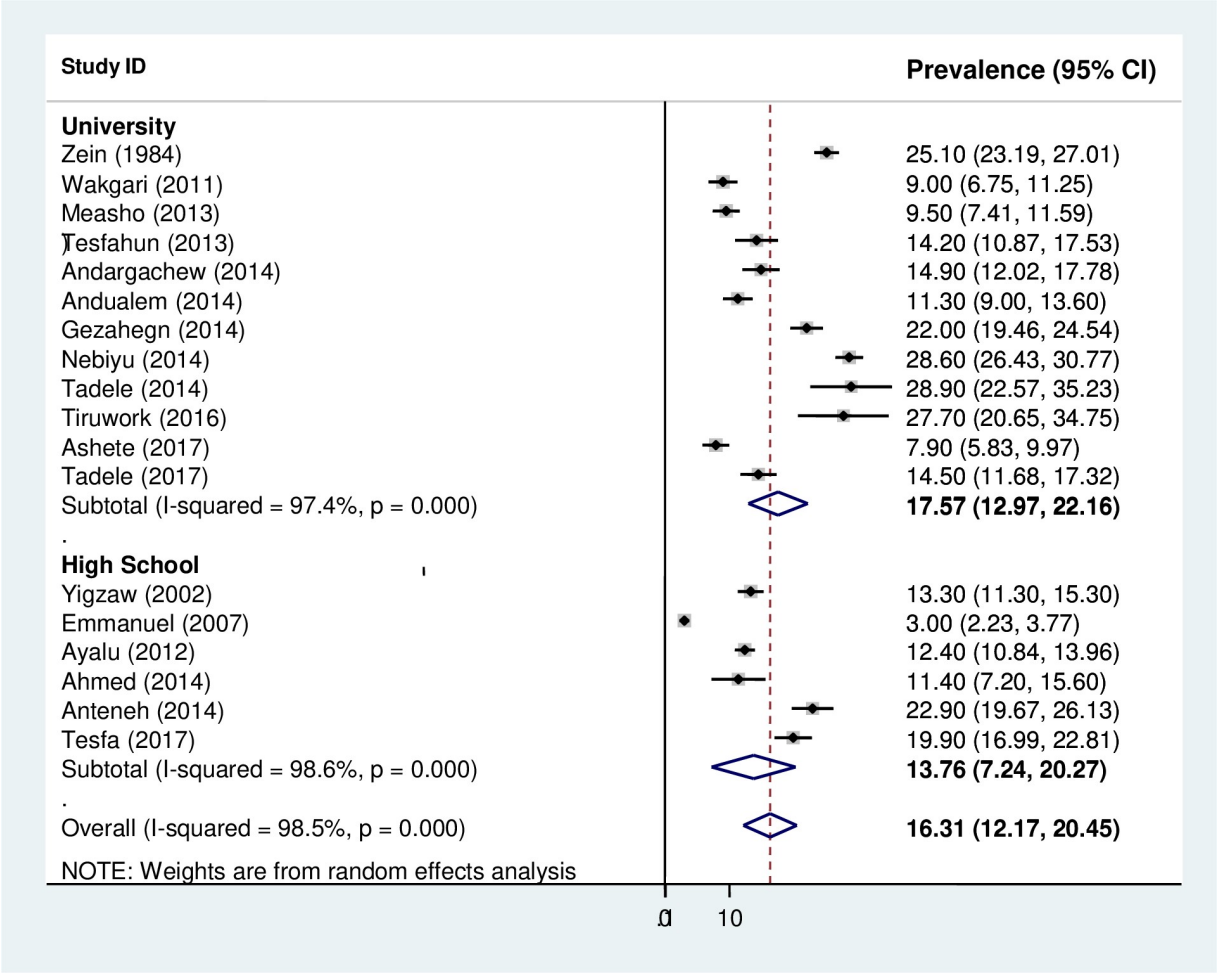
Forest plot of the pooled prevalence of cigarette smoking among high school and university students across regions in Ethiopia.

The findings from the subgroup analysis showed that the highest and lowest cigarette smoking was observed among university students 17.35% (95% CI: 12.97, 22.16) and high school students 13.76% (95% CI: 7.24, 20.27), respectively (Fig 4).

**Fig 5:**
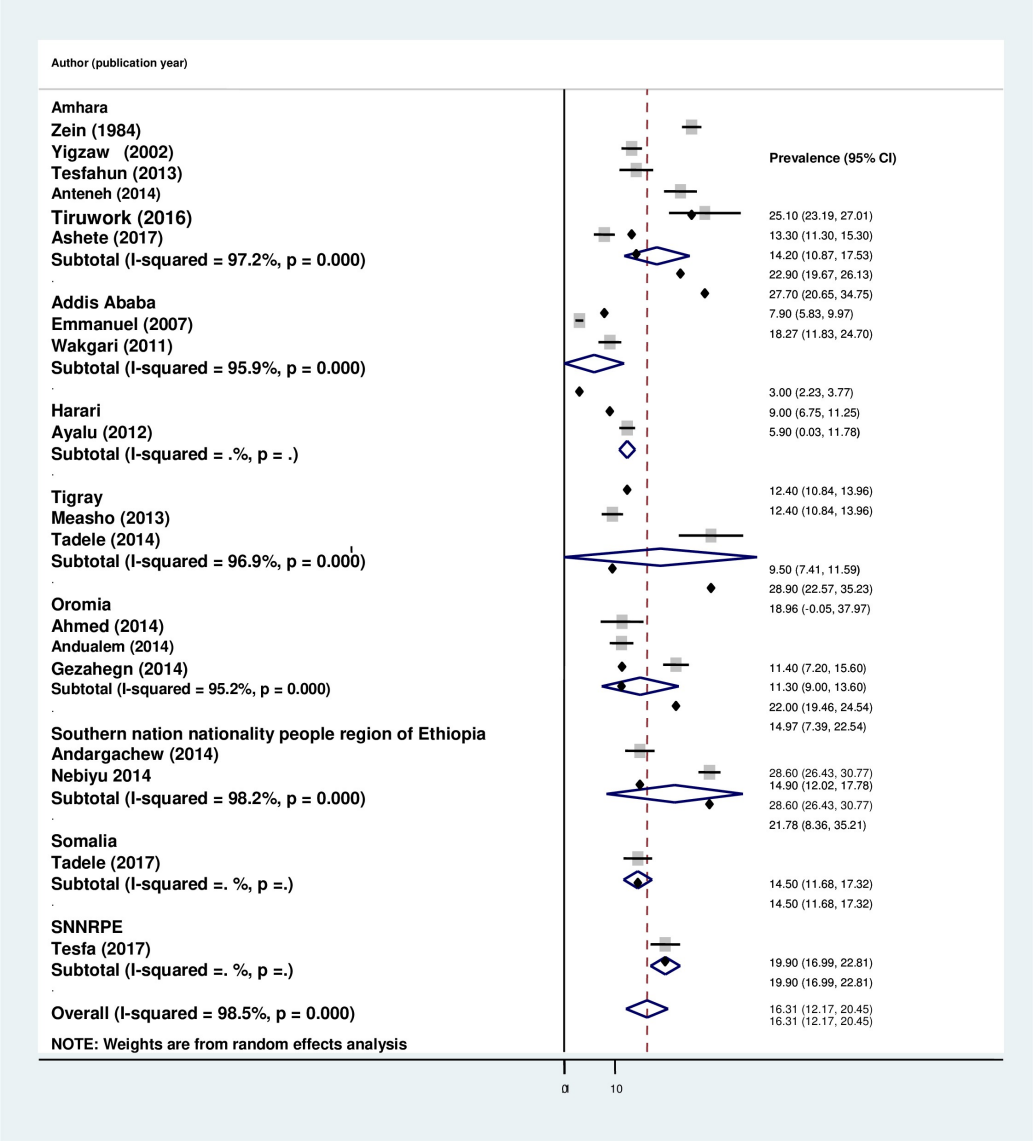
Forest plot graph indicates, subgroup analysis of cigarette smoking among the region, in high school and university students in Ethiopia.

Similarly, the regional subgroup analysis result revealed the pooled prevalence of smoking from highest to lowest was [20.11% (95% CI: 11.39, 28.84)] in Ethio-Somalia and Harari region, [18.96% (95% CI: -0.03, 38.01)] in Tigray region, [17.35% (95% CI: 13.21, 21.49)] in South Nation Nationality and People of Ethiopia (SNNPE), [15.34% (95% CI: 10.84, 19.83)] in Amhara region, [14.98% (95% CI: 7.37, 22.55)] in Oromia region, and [5.9% (95% CI: 0.02, 11.79)] in Addis Ababa region (Fig 5).

### The linear trend of cigarette smoking status of students in Ethiopia

**Fig 6:**
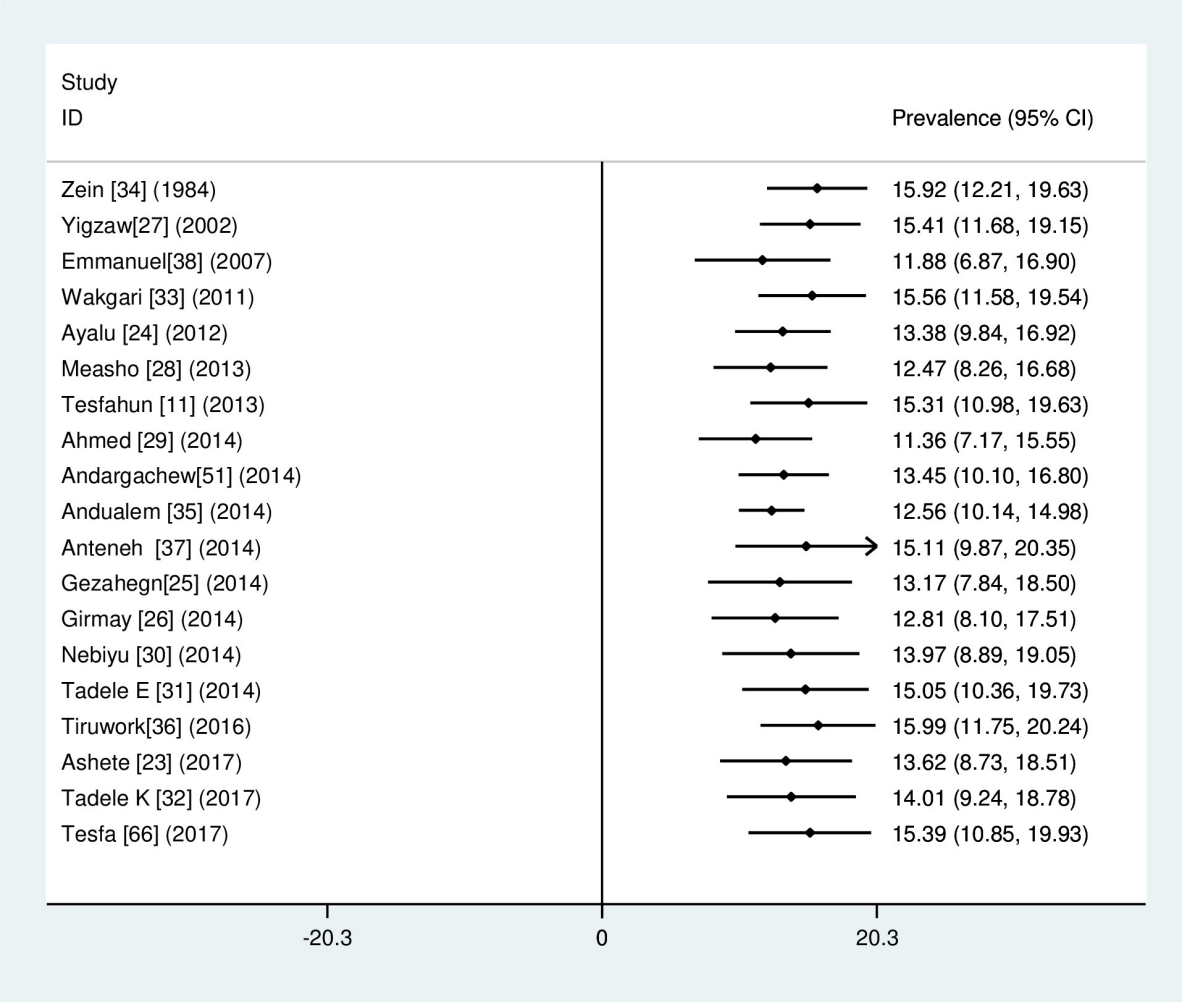
Linear trend of cigarette smoking among high school and university students in Ethiopia.

The cumulative univariate meta-analysis on cigarette smoking status among high school and university with the year of 1984–2017 was performed. The result from cumulative univariate meta-analysis showed the trend in prevalence estimates of cigarette smoking status among high school and university over time. The finding revealed that there is more or less constant trend (Fig 6).

**Fig 7:**
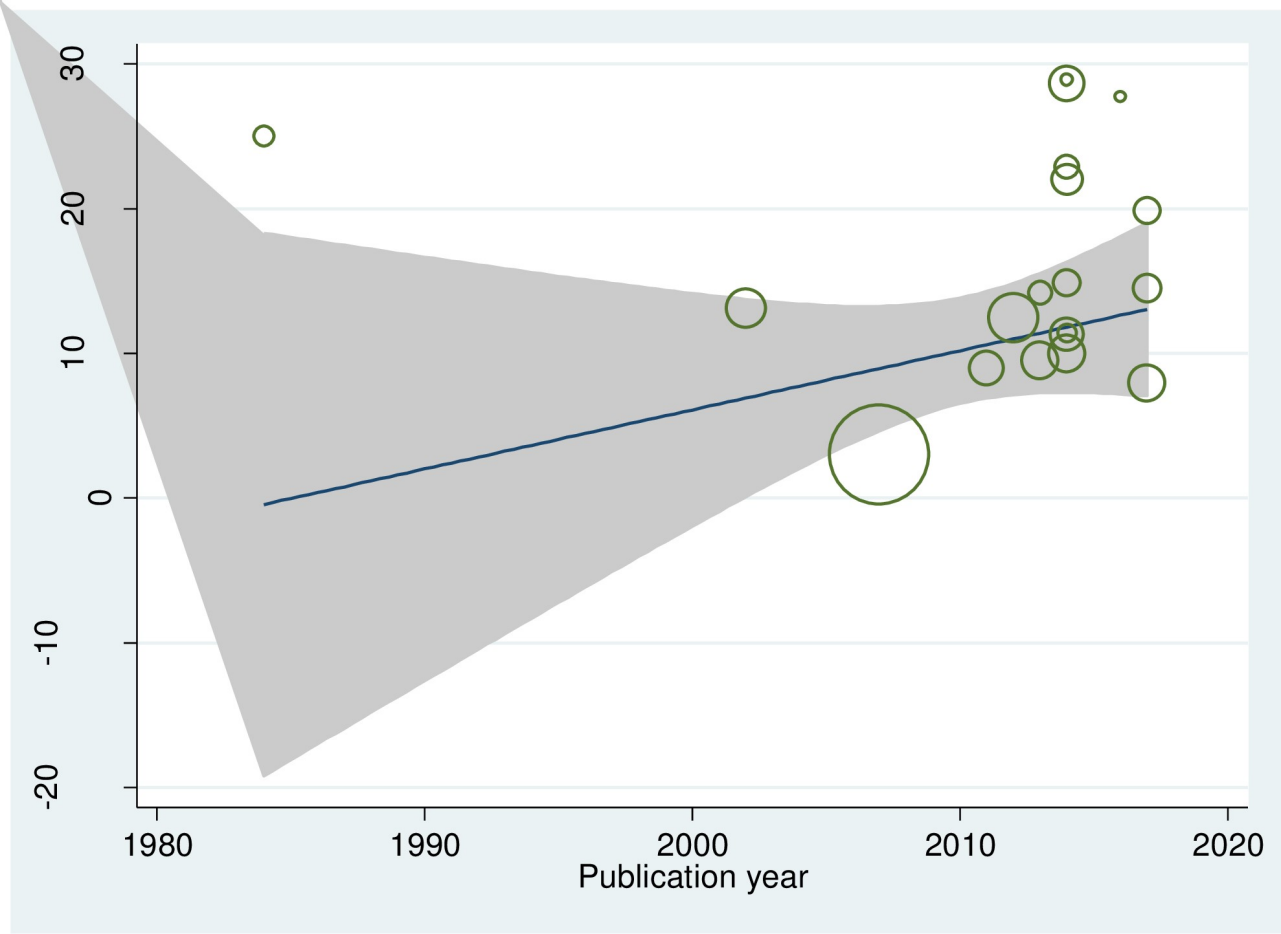
Describe the univariate meta-regression using bubble plot cigarette smoking among high school and university students in Ethiopia.

The univariate meta-regression using bubble plot was also performed. The bubble plot figure indicates that the trend was slight increment (Fig 7).

### The effect of peer pressure on cigarette smoking status

**Fig 8:**
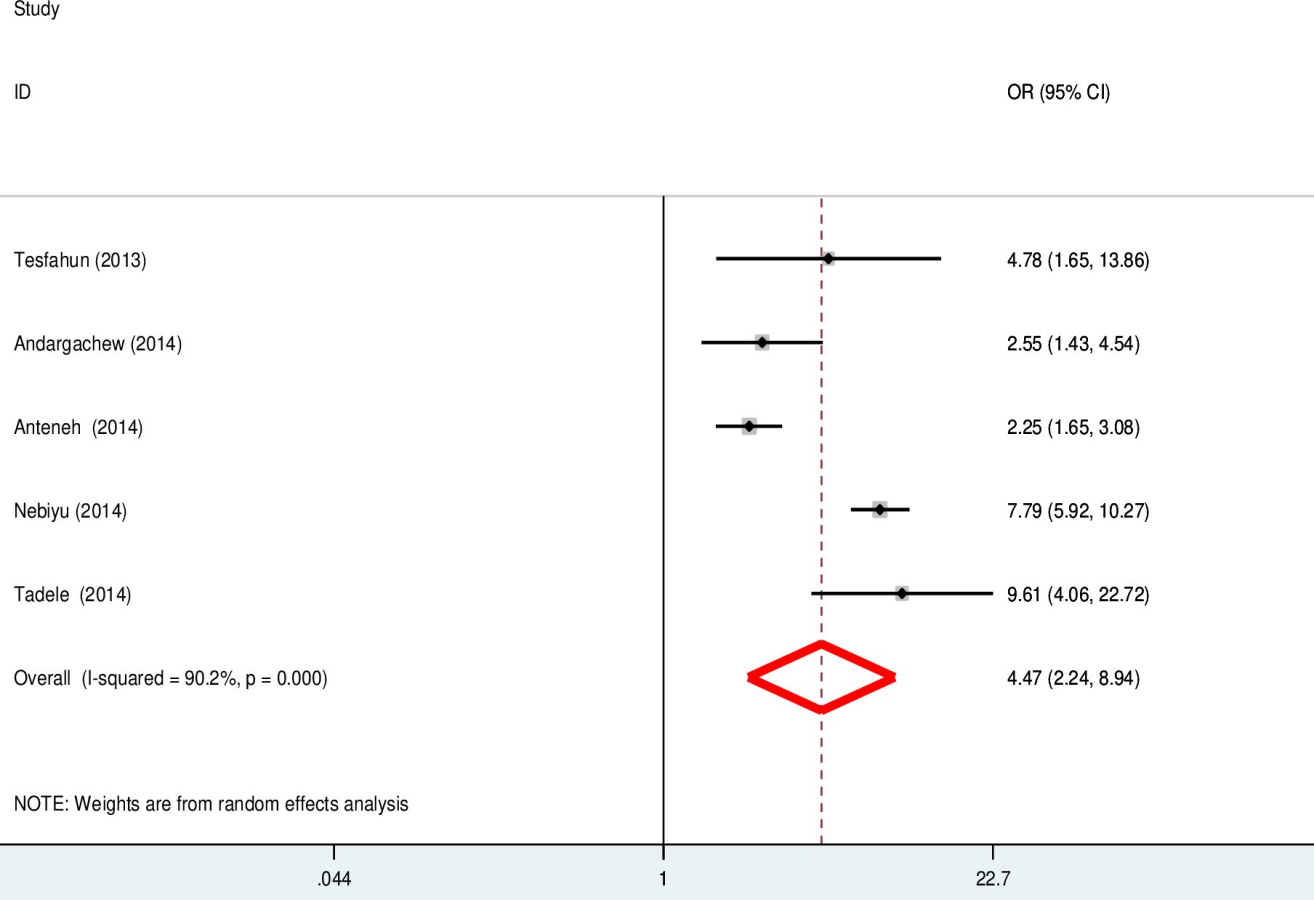
Describe the pooled odd ratio of the impact of peer pressure on cigarette smoking status among university and high school students in Ethiopia.

Five of the 19 included studies reported the effect of peer pressure on cigarette smoking. From this, three studies [11, 30, 37] showed a positive effect of peer pressure on cigarette smoking, while the other two studies [31, 51] showed no relationship between peer pressure and cigarette smoking. However, the aggregated meta-analysis revealed a higher odds of cigarette smoking among students who experienced peer pressure than those who didn’t (OR: 2.68, 95% CI: 2.37, 3.03) (Fig 8).

## Discussion

Cigarette smoking has major health and social consequences, and it reduces the educational performance of students [52, 53]. This systematic review and meta-analysis, therefore, was conducted to assess the pooled prevalence of cigarette smoking and its association with peer pressure among high school and university students in Ethiopia. Accordingly, the pooled prevalence of cigarette smoking among Ethiopian high school and university students was 15.92%. This finding is lower than a study conducted among students in South Africa which reported a prevalence of 16.9% [50]. Conversely, the current reported pooled prevalence of cigarette smoking was higher than a study conducted among government and private schools and college students in Bengaluru, India (12.8%) [54] and amongst university students in Iran (13.8%) [55].

In this review, the pooled prevalence of cigarette smoking was lower than a study finding observed among Kenyan secondary school students (38.6%) and Cameroon university students (93.1%) [56, 57]. In addition, our finding was slightly lower than a study conducted among high school students in Shiraz- Iran (19.7%) [58]. This might be due to the difference between sample size and socio-demographic nature of the two study populations. There is also cultural variation among the study communities. Moreover, the higher prevalence of cigarette smoking in the current study could be due to the dominance of male participants as evidence suggests that males tend towards different types of substance abuse than females [59, 60].

Similarly, the current pooled prevalence of cigarette smoking is also lower than a systematic review conducted in Africa [50] and the Middle East [61]. This variation might be due to the differences in the study period and sample size between these two studies. In addition, the previous review was conducted only among university students, while the current review included both high school and university students.

The current review also considered subgroup analysis to appreciate the variability or heterogenic characteristics of the included studies. Accordingly, a higher prevalence was observed among university students (17.35%) than high school students (12.77%). This could be because most high school students live with their families which may limit them from cigarette smoking because of parental control. Additionally, in most cases, students during their high school time live with families and that may not encourage smoking cigarette. On the contrary, when they join to the university, almost all students become independent of their family supervision. This independency and pressure from their friends increases the proportion of students who smokes cigarette [62]. Educational institutions can be a challenging environment and everyone copes with stress in different ways [17]. Moreover, as students enter to university, they start a new life away from their families in a different and strange environment which can contribute to their behavior or involvement in substance abuse like cigarette smoking [55]. Evidence also supports that as the level of education increase, the proportion of smoking increases [63, 64].

A subgroup analysis by regions of the country also showed a higher prevalence of cigarette smoking among universities in other category (i.e., Harar region, Somalia region and Oromia region). This finding might be due to typical local practices of substances like cigarette and khat in these regions. Therefore, the government, school management, local communities and other concerned bodies need to implement school-based intervention programs in order to reduce the pooled prevalence of cigarette smoking.

Students who felt peer pressure were more likely to smoke cigarette than those who had no peer pressure. This finding was similar to a study conducted in Kenyan students and Shiraz- Iran [57] where peer pressure was found to have a significant (positive) effect on the likelihood of cigarette smoking [56, 58]. Peer group pressure is widely known as a decisive factor which affects the early onset of experimentation with tobacco and the individual’s subsequent willingness to continue smoking [16]. Similarly, other systematic reviews state the most common factors influencing students’ smoking status was having smoker friends [55, 65]. Therefore, the school management needs to implement youth association focusing on counseling and rehabilitation service for to seize students already practicing smoking and also those who are not practicing yet now.

### Strengths and limitations of the study

This review has several strengths including: this review focus on the adolescent and young adult populations who are vulnerable to initiating substance use/abuse behaviors. In addition, this review rigorous adherence to the PRISMA checklist which improves its quality for the readers. Moreover, this finding will give an insight into developing a health promotion policy for the country. Whereas, on top of the above strength, this review has the following limitations: This review included studies that were published only in English language which may limit the number of studies that were reported in other languages. Moreover, the other limitation of this review was the risk of self-report bias introduced from the original studies included in the review. On top of these the protocol of this manuscript was not registered online before conducting it.

## Conclusions

This systematic review and meta-analysis indicate that the prevalence of cigarette smoking among Ethiopian high school and university students was high. More than one sixth of the high school and university students smoke cigarettes. This higher cigarette smoking proportion of students was influenced by peer pressure. Variations were also observed in the prevalence of cigarette smoking by different regions in the country. Therefore, school-based intervention programs aimed at prevention of cigarette smoking is recommended. In particular, educational programs on how to resist and handle peer pressure are essential to prevent cigarette smoking among high school and university students in Ethiopia.

## Supporting information

S1 Table PRISMA 2009 checklist. (DOC)

S2 Table Searches for databases. (DOCX)

S3 Table Data extraction tools Smoke. (XLSX)

S4 Table Quality assessments. (DOCX)

S5 Table NIH quality assessments. (DOCX)

S6 Table Risk of bias for each study. (XLSX)

## Abbreviations

CI: Confidence Interval
HIV: Human Immune Deficiency Virus
OR: Odd Ration
PRISMA: Preferred Reporting Items for Systematic Reviews and Meta-Analyses
SE: Standard Error
SNNPR: South Nation and Nationalities People of the Region
RR: Relative Risk
WHO: World Health Organization
