# Supplementary material for: The impact of peer pressure on cigarette smoking among high school and university students in Ethiopia: A systemic review and meta-analysis
Source: PLoS One. 2019 Oct 11;14(10):e0222572. doi: 10.1371/journal.pone.0222572 (PMC6788683; doi:10.1371/journal.pone.0222572)
Supplement: S2 Table — (DOCX) [file pone.0222572.s002.docx]

| **Databases** | **Searching terms** | **Number of studies** |
| --- | --- | --- |
| **Google scholar** | "prevalence" and "determinants" or "associated factors " and "high school" or "university" and "students" or " cigarette" and "Ethiopia" | **108** |
| **MEDLINE/ PubMed** | The reviewer used the following keywords “prevalence”, ("cigarette smoking"[MeSH Terms] OR ("cigarette"[All Fields] AND "smoking"[All Fields]) OR "cigarette smoking"[All Fields]) AND substance[All Fields]) AND (high[All Fields] AND ("schools"[MeSH Terms] OR "schools"[All Fields] OR "school"[All Fields]) AND ("universities"[MeSH Terms] OR "universities"[All Fields] OR "university"[All Fields])) AND ("students"[MeSH Terms] OR "students"[All Fields]) AND ("Ethiopia"[MeSH Terms] OR "Ethiopia"[All Fields]) | **43** |
| **From other databases** |  | **368** |
| **Total retrieved articles** |  | **535** |
| **Final full text relevant to our review** |  | **30** |
