# Supplementary material for: The impact of peer pressure on cigarette smoking among high school and university students in Ethiopia: A systemic review and meta-analysis
Source: PLoS One. 2019 Oct 11;14(10):e0222572. doi: 10.1371/journal.pone.0222572 (PMC6788683; doi:10.1371/journal.pone.0222572)
Supplement: S4 Table — (DOCX) [file pone.0222572.s004.docx]

| **Articles** | **Selection 1)**  **a=b=1, c=d=0** | **Selection 2)**  **a=1, b=0** | **Selection 3)**  **a=1, b=0, c=0** | **Selection 4)**  **a=2, b=1, c=0** | **Comparability 1) a=b=1** | **Outcome 1)**  **a=b=2, c=1, d=0** | **Outcome 2)**  **a=1, b=0** | **Score** |
| --- | --- | --- | --- | --- | --- | --- | --- | --- |
| Wakgari D | a) | a) | a) | c) | a) b) | b) | a) | 8 |
| Measho G. et al | a) | a) | a) | a) | a)b) | c) | a) | 9 |
| Ahmed Yasin M. et al. | a) | a) | b) | a) | a) b) | a) | a) | 9 |
| Girmay T | a) | a) | a) | b) | a) b) | c) | a) | 8 |
| Gezahegn T et al | a) | a) | a) | a) | a) b) | b） | a) | 10 |
| Ayalu A, Reda et al | a) | a) | a) | a) | a) b) | b) | a) | 10 |
| Andargachew K et al | a) | a) | a) | b) | a) b) | b) | a) | 9 |
| Tadele Kinati B et al | a) | a） | a) | b) | a) b) | c) | a) | 8 |
| Tadele E | a) | a) | b） | a) | a) b) | b) | a) | 8 |
| Yigzaw Kebede | a) | a) | a) | a) | a) b) | c) | a) | 9 |
| Ashete A, et al | b) | a) | a) | a) | a) b) | b) | b) | 9 |
| Emmanuel R et al | a) | a) | a) | a) | a) b) | d) | a) | 8 |
| Nebiyu D, et al | a) | a) | b) | a) | a) b) | b) | a) | 9 |
| Anteneh MB, et al | a) | a) | a) | a) | a) b) | b) | a) | 7 |
| Zein ZA et al | a) | a) | b) | b) | a) b) | c) | a) | 7 |
| Andualem D, et al | a) | b) | a) | b) | a) b) | b) | a) | 8 |
| Tiruwork T, et al | a) | a) | a) | c) | a) b) | a) | a) | 9 |
| Tesfa M, et al | a) | a) | a) | c) | a) b) | c) | a) | 7 |
| Tesfahun A, et al | a) | a) | a) | c) | a) b) | b) | a) | 8 |

**NB: high quality score > 7**
